# Health impacts of climate change and geopolitics: a call for papers

**DOI:** 10.2471/BLT.20.251934

**Published:** 2020-03-01

**Authors:** Payao Phonsuk, Rapeepong Suphanchaimat, Walaiporn Patcharanarumol, Diarmid Campbell-Lendrum, Viroj Tangcharoensathien

**Affiliations:** a International Health Policy Program, Ministry of Public Health, Nonthaburi 11000, Thailand.; b Department of Environment, Climate Change and Health, World Health Organization, Geneva, Switzerland.

Despite the severe impacts of climate change on human health, socioeconomic development and the natural environment, the United Nations (UN) Framework Convention on Climate Change has failed to reach its full potential. Although State Parties have endorsed one protocol and two agreements as part of the convention, progress has been slow. In 2018 alone, global carbon dioxide emissions increased by 2.7%, from 36.1 to 37.1 billion tonnes,^1^ one of the highest annual increases in the last ten years. This trend has already jeopardized the possibility of reaching the UN Intergovernmental Panel on Climate Change goal of limiting global warming to 1.5 °C, of reducing carbon dioxide emissions by 45% by 2030 and reaching a net-zero by 2050.

The main drivers of climate change cause direct health effects. Fossil fuel combustion is responsible for approximately two thirds of exposure to ambient air pollution from fine particulate matters (PM_2.5_). Such air pollution is one of the major risk factors for cardiopulmonary diseases and lung cancer,^2^ as well as other noncommunicable diseases, the world’s leading causes of death and the largest drain on health system resources.

Climate change also affects agriculture because of changing rainfall patterns and drought, rising temperatures and variability in seasonality, which hampers progress on the sustainable development goal of ending hunger though food security and improved nutrition.^3^ Nonetheless, agriculture contributes to climate change as well, through emissions of greenhouse gas, the conversion of non-agricultural land into arable land, resulting in deforestation and loss of biodiversity.

Addressing the causes of climate change can benefit human health and ecosystems.^4^ The 2015 Paris Agreement has set the goal to limit temperature rise to below 2.0 °C (ideally 1.5 °C) globally. However, to date no country is performing satisfactorily, as measured by the Climate Change Performance Index.^5^

The effects of climate change can also interact with one or several aspects of geopolitics; however, neither are adequately addressed by the global health agenda. For example, China has constructed several hydropower dams on the Mekong River that have led to ecological and environmental changes that affect the livelihood of the people in the Mekong sub-region, both within and outside China.^6^ Similarly, negative impacts of the Grand Ethiopian Renaissance Dam on the Nile River in Ethiopia are reported.^7^ A study in Sudan’s Er Roseires Dam shows mixed results of positive impacts, such as fishery, farming and collection of wood and fruits, combined with negative impacts of increased humidity and health problems.^8^

Political conflicts in fragile states, coupled with drought and famine because of climate change, contribute to international and domestic migration. Population displacement negatively affects the physical and mental health of the displaced, particularly the most vulnerable groups.

Evidence shows that geopolitics has influenced the allocation of foreign assistance.^9^ Although foreign direct investments boost local economies, investment in health services raises concern over the affordability and widening inequity of access to health services, particularly in the context of weak government regulatory capacity.

In addition, countries with high economic and political power often play a critical role in shaping the global health agenda, to safeguard their national interests, rather than to promote global health more equitably. Reforming global governance for health and improving the capacity of low- and middle-income countries to negotiate and safeguard their interests is therefore necessary.^10^

The *Bulletin of the World Health Organization* will publish a theme issue on the impacts of climate change and geopolitics, and their interlinkages with human health. Specifically, the issue will explore how health systems in low-resource settings mitigate and adapt to these impacts; how the UN Member States, civil society organizations and citizens can foster commitments towards various international instruments on climate change; the role of monitoring and reporting and civil society organizations in holding governments accountable for addressing climate change and influencing geopolitical forces so health is promoted and protected.

We particularly encourage manuscripts that identify effective and feasible policy interventions and national and global governance for health that can contribute to protecting and promoting health and enhancing health equity.

The deadline for submission is 15 June 2020. Manuscripts should be submitted in accordance with the *Bulletin’s* guidelines for contributors (available at: http://www.who.int/bulletin/volumes/98/1/20-960120/en) and the cover letter should mention this call for papers.
